# Urgent orotracheal intubation in critically ill patients

**DOI:** 10.1186/cc9572

**Published:** 2011-03-11

**Authors:** M Hernández Bernal, JJ Manzanares Gomez, C Soriano Cuesta, A Agrifoglio Rotaeche, J Figueira, J López, M Jimenez Lendinez

**Affiliations:** 1 La Paz University Hospital, Madrid, Spain

## Introduction

The aim of this study is to analyze the incidence of difficult intubation, and likewise characteristics, complications and mortality of urgent orotracheal intubation (OTI) in critically ill patients.

## Methods

An observational, descriptive and prospective study. We analyze the impact of difficult OTI, morbidity and mortality in urgent OTI, in the noncoronary ICU of a third-level university hospital in Madrid. We collected all OTIs during the period of 1 year. Demographic data, blood pressure and O_2 _saturation with pulsioximetry, before and after OTI, indications, type of technique, medication administrated, place where the technique was performed, and complications were collected.

## Results

Patients: 277. OTIs: 305. Average attempts: 1.15 (SD: 0.41). Sex: male (M): 197 (64.6%), female (F): 108 (35.4%). Age: 56 years (15 to 87). Indications for OTI: low level of consciousness: 103 (34%), excessive work of breathing: 88 (29%), airway protection: 58 (19%), poor secretion management: 44 (14.4%), endotracheal tube change: 29 (9.5%), combative patient: 27 (8.8%), autoextubation: 6 (2.1%), glottis or laryngeal edema: 5 (1.7%), others: 6 (2%). Two or more indications agreed in 36%. Place technique was performed: ICU: 172 (56.4%), Emergency Department (ED): 85(27.9%), hospital ward: 29 (9.5%), burn unit: 16 (5.2%), others: 3 (1%). Complications: 113 (37%): hemodynamic deterioration: 72 (23.6%), hypoxemia: 22 (7.2%), esophageal intubation: 5 (1.6%), selective bronchial intubation: 4 (1.3%), bronchoaspiration: 4 (1.3%), impossible OTI: 3 (0.9%), others: 3 (0.9%). Difficult and impossible OTI: 7 (2.3%): difficult OTI: 4 (1.3%), impossible OTI: 3 (0.98%). Average age: 52 years (38 to 81). Sex: M: 3 (42.8%), F: 4 (57.2%). Place technique was performed: ICU: 3 (42.9%), ED: 2 (28.5%), hospitalization ward: 1 (14.3%), burn unit: 1 (14.3%). Average attempts: 4.5 (SD 0.5). Total mortality of the study: 3 (0.98%).

## Conclusions

In our study, difficult intubation rates were lower than those reported in other series, so it is remarkable the low mortality of the series, less than 1%, which was determined by hemodynamic deterioration after the technique and not associated with the procedure. In view of the results it is advisable to carry out predictive tests, taking into account the characteristics of the critical patients who require urgent intubation, to provide technical difficulties in carrying out the process and anticipate the preparation of necessary materials before starting sequence intubation; likewise, new systems have access to the airway for risk.
